# Th17 Cells in Immunity and Autoimmunity

**DOI:** 10.1155/2013/986789

**Published:** 2013-12-26

**Authors:** Simone Kennedy Bedoya, Brandon Lam, Kenneth Lau, Joseph Larkin

**Affiliations:** Department of Microbiology and Cell Science, University of Florida, P.O. Box 110700, Museum Road Building 981, Gainesville, FL 32611, USA

## Abstract

Th17 and IL-17 play important roles in the clearance of extracellular bacterial and fungal infections. However, strong evidence also implicates the Th17 lineage in several autoimmune disorders including multiple sclerosis, psoriasis, rheumatoid arthritis, inflammatory bowel disease, systemic lupus erythematosus, and asthma. The Th17 subset has also been connected with type I diabetes, although whether it plays a role in the pathogenicity of or protection from the disease remains a controversial issue. In this review we have provided a comprehensive overview of Th17 pathogenicity and function, including novel evidence for a protective role of Th17 cells in conjunction with the microbiota gut flora in T1D onset and progression.

## 1. Introduction

Antigen presenting cells activate naïve T lymphocytes by presenting specific antigens, and by providing the necessary costimulatory signals and cytokine environment [1]. T lymphocytes bearing T cell receptor (TCR) specificity towards the presented antigen subsequently undergo clonal expansion and mediate effector functions largely dictated by the stimulatory and environmental clues provided [2]. In a past classical model, CD4+ effector T cells were assigned to either the Th1 or Th2 subset, each with its own distinct cytokines, transcription factors, and functions [3]. Th1 cells produce IFN*γ* and are regulated by IL-12 through the transcription factor Tbet, while Th2 cells produce the cytokines IL-4, IL-5, and IL-13 and are regulated by the transcription factor GATA3. Th1 cells are associated with protection against intracellular pathogens, and T lymphocytes bearing the Th2 phenotype regulate humoral immunity, and are involved in the protection against extracellular pathogens [4]. Having established a role for Th1 and Th2 cells within the context of immune defense against microorganisms, the Th1/Th2 paradigm was then utilized to garner insight into the onset and progression of autoimmune disorders. The goal of this review is to explicate how limitations of the Th1/Th2 paradigm in the context of autoimmunity led to the discovery of the Th17 phenotype, and to examine the implications of the Th17 phenotype within the context of several autoimmune disorders, including T1D.

## 2. A Shift in Focus to the Th17 Phenotype

The experimental autoimmune encephalomyelitis (EAE) model of multiple sclerosis (MS) provided the first clues to the possibility that other T cell effector functions, beyond those attributed to the Th1 and Th2 subsets, could be contributing to the onset and progression of autoimmune disorders. Under the previously existing dogma, IL-12 and henceforth Th1 cells and IFN*γ* were thought to be central in disease progression and severity. However, several studies noted irregularities in this theory as mice models including IFN*γ*−/−, IFN*γ*R−/−, IL-12*β*R−/−, and IL-12p35−/− mice showed an exacerbation and progression of the disease [5–10], an exacerbation that was downregulated and protected with a functional Th1 pathway intact [5–7].

A major development came in 2003 by Cua et al. when the critical cytokine in murine EAE was elucidated as interleukin-23 (IL-23), a heterodimeric cytokine composed of the p40 and p19 subunits [11, 12]. Previous work had shown that blocking IL-12 or the IL-12p40 subunit in animal models conferred protection from EAE [13–17]; however, the p40 subunit is shared by both IL-12 and IL-23 [12]. Cua et al. showed that mice with a deleted p19 subunit specific for IL-23 were protected from EAE development. Mice lacking the p35 subunit specific for IL-12, however, were still susceptible to EAE development. A study by Murphy and colleagues in 2003 followed shortly that also described a protective role for IL-12 and IFN*γ* in models of collagen-induced arthritis (CIA), another disease which was previously thought to follow the Th1/Th2 model as well [18]. Murphy et al. determined that IL-23 promoted a subset of IL-17-producing CD4+ T cells, which in turn furthered CIA disease progression [18].

These and subsequent studies in 2005 led to the realization that there was a novel subset of T helper cells distinct from the classical Th1/Th2 paradigm [19–21]. This novel subset, termed Th17 for its production of interleukin-17 (IL-17), involves a combination of cytokines, transcription factors, and immunological functions that make it distinct from both T helper 1 and 2 cells.

## 3. The Th1/Th2 Paradigm is Insufficient for Type 1 Diabetes

The pivotal research studies which implicated Th17 cells in the roles previously assigned to Th1 cells regarding autoimmunity also prompted an interrogation of the Th17 phenotype within the context of T1D. Under the classic Th1/Th2 paradigm, IFN*γ*-producing Th1 cells were strongly implicated as a major contributor to T1D progression [22–24], while Th2 T lymphocytes were identified as having a protective role [25]. However, studies have shown that the loss of either the IFN*γ* receptor or IFN*γ* production failed to prevent the spontaneous development of T1D in nonobese diabetic (NOD) mice [26, 27], while another study showed that IFN*γ* induction restored normoglycemia [28]. Moreover, it was shown that IL-4 deficiency did not exacerbate disease [29], calling into question the protective role of Th2 lymphocytes. Together these studies made it clear that the Th1/Th2 paradigm was insufficient to explain the immunopathogenic events leading to autoimmune diseases such as T1D.

The resulting evidence from both human and rodent studies regarding the role of Th17 cells and IL-17A production in the onset and development of T1D has been conflicting. Monocytes derived from T1D patients spontaneously induce Th17 cells [30], and it has been shown that Th17 cell inhibition was sufficient to regulate T1D in the NOD mouse model [31]. Conversely, it has been shown that Th17 cells delay T1D in NOD mice treated with mycobacterial adjuvant [32]. IL-17-producing gamma delta T cells have also been shown to have a protective role in the NOD model of spontaneous T1D [33]. The controversial nature of IL-17 production in relation to T1D is likely due to the fact that IL-17 is in actuality produced by a spectrum of T cell phenotypes possessing both varied and distinct effector functions.

It has become clear that the Th17 lineage is not a single distinct lineage, but rather encompasses several T lymphocytes which all produce IL-17A, but possess varied effector functions. Although IL-17-producing cells have been identified in the onset and progression of several autoimmune disorders, we now know that not all IL-17-producing T lymphocytes are pathogenic [34]. Strikingly, researchers have shown that IFN*γ*+IL-17+ T lymphocytes, which originate from IL-17-producing Th17 cells, are closely associated with pathogenicity [35]. This finding is likely to be significant in the understanding of T1D, as Th17 differentiated CD4+ T lymphocytes are incapable of mediating T1D in the inducible NOD.scid model prior to acquisition of the IFN*γ* effector function [36, 37]. While this may suggest that IFN*γ* mediates T1D onset, it is more likely that onset is mediated by an effector function present in this population of IFN*γ*+IL-17+ cells, but absent in the IFN*γ*+ and IL-17+ single cytokine-producing populations.

Notably, the discovery of the Th17 phenotype was closely paralleled by the characterization of yet another CD4+ T cell lineage, the Foxp3+ regulatory T cells (Treg), which are critical in the prevention of immune responses directed against self-antigens [38, 39]. Although generally associated with protection from inflammation and autoimmunity, Tregs have recently been shown to be highly plastic in an inflammatory cytokine environment. Studies have shown in both murine models and humans that Tregs stimulated *ex vivo* with IL-6 or IL-1*β* can be induced to produce IL-17 [40, 41]. For example, it has been shown that Tregs (identified as “ex-Tregs”) that acquire the IL-17 effector function can mediate T1D [42]. Tregs treated with IL-6 have also been shown to lose their ability to protect mice from a lupus-like disease [43]. ROR*γ*T+Foxp3+ and IL-17+Foxp3+ T cells have also been identified *in vivo* and are unable to control inflammation [44, 45]. These IL-17+Foxp3+ T cells have also been identified in the lamina propia of Crohn's Disease patients but not in those from healthy controls [45].

## 4. Differentiation of Th17 Cells 

**Figure 1:**
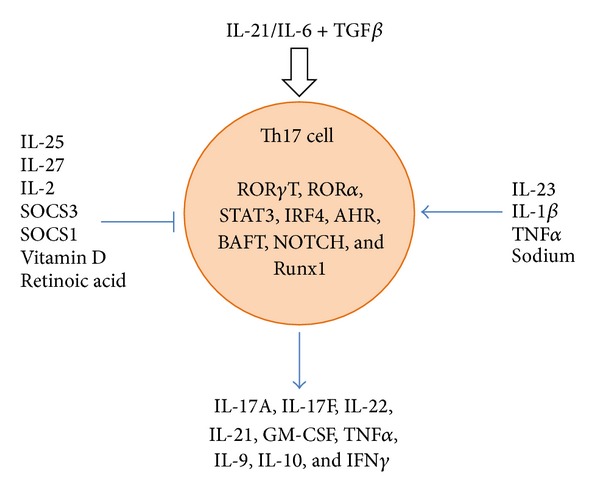
The world according to Th17 cells. Th17 cell induction occurs through combination of IL-6 and TGF*β*, or IL-21 and TGF*β*—in the absence of IL-6. The Th17 lineage produces cytokines which include IL-17A, IL-17F, IL-22, IL-21, GM-CSF, TNF*α*, IL-9, IL-10, and IFN*γ*. This population is enhanced through IL-23, IL-1*β*, TNF*α*, and high levels of sodium. Conversely, IL-25, IL-27, IL-2, SOCS3, SOCS1, Vitamin D, and retinoic acid serve to negatively regulate Th17 cells.

Th17 differentiation, survival, and expansion rely on a variety of cytokines and transcription factors that work in concert to drive the induction of increased Th17 numbers and to also suppress the induction of other lineages of T helper cells (Figure 1). Transforming growth factor *β* (TGF*β*) in synergy with IL-6 have been described as the central factors involved in generating *de novo* Th17 cells [46–49]. TGF*β* has also been implicated in the differentiation of Foxp3+ Tregs [50]; however, it seems that TGF*β* favors Th17 induction in an inflammatory environment [46, 49, 51]. IL-6 can also act to induce expression of IL-21, which then acts as a positive autocrine feedback loop, inducing more IL-21 production and upregulating IL-23 receptor alpha chain expression (IL23R) [52, 53]. In addition, it has been shown that Th17 cells can be induced by IL-6, IL-1*β*, and IL-23 independent of TGF*β* [54]. Notably, these “alternatively- derived” Th17 cells have been described as being more pathogenic than those derived from IL-6 and TGF*β* alone [34].

Although IL-23 was originally thought to be critical in the *de novo* generation of the Th17 lineage, it was revealed that IL-23 acts to sustain the phenotype subsequent to the upregulation of IL-23R. IL-23 is necessary for Th17 lineage expansion and survival as p19−/− mice may still be capable of producing Th17 cells, but without IL-23 the population is not able to expand and survive [48]. In more recent studies, it has been shown that IL-23 signaling significantly contributes to the pathogenicity of the Th17 subset [34]. In addition to Th17 lineage amplification, IL-21 has also been shown in an alternative pathway model that, together with TGF*β* and in the absence of IL-6, can also induce generation of Th17 cells [55]. The process of Th17 differentiation has been found to be amplified through many different factors including TNF*α* and IL-1*β* [48, 56]. Differentiated Th17 cells produce a variety of cytokines including IL-17A, IL-17F, IL-21, IL-22, GM-CSF, IL-9, IL-10, and IFN*γ*, which vary depending on the cytokine milieu in which the differentiation occurs [57]. IL-17A is considered the primary effector cytokine of the Th17 lineage, but it also shares a 55% identity to IL-17F [58]; both are capable of forming homodimers or combining to form heterodimers [59, 60]. In relation to T1D, one of the more interesting cytokines associated with the Th17 lineage is GM-CSF, as it has been shown that GM-CSF production can mediate the induction of tolerogenic, protective dendritic cells [61, 62]. It would be worth investigating whether differences in GM-CSF production by IL-17-producing T lymphocytes could account for the published discrepancies in Th17-mediated T1D protection or pathogenicity.

CD4+ T lymphocyte lineages are regulated by specific transcription factors. Whereas Th1 and Th2 lineages are modulated by the transcription factors, T-bet, and GATA3, respectively [63]; the central modulator of the Th17 lineage is the orphan nuclear receptor *γ* (ROR*γ*T) [64]. ROR*γ*T directs the transcription of genes encoding IL-17A and IL-17F among others; however, ROR*γ*T deficiency does not completely abrogate Th17 differentiation. It was found that ROR*α* also contributes a similar role in the differentiation of naïve CD4+ T cells, and both are capable of being induced by TGF*β* and IL-6 [65].

Other transcription factors involved in directing naïve CD4+ T cells toward Th17 differentiation include STAT3, interferon regulatory factor 4 (IRF4), the aryl hydrocarbon receptor (AHR), NOTCH, and BATF [66–72]. STAT3 can be activated by IL-6 and IL-23, and regulates the expression of ROR*γ*T, as STAT3 deficiency impairs ROR*γ*T expression and leads to elevated expressions of T-bet and Foxp3 [67]. It has also been suggested that STAT3 is able to bind directly to the *IL17* promoter, thereby further enhancing a cell's commitment to the Th17 lineage [66]. It was shown that IRF4 was significant in Th17 differentiation when IRF4−/− mice were incapable of generating Th17 cells or IL-22 production and were also resistant to developing EAE [68]. IRF4−/− mice had normal STAT3 levels, but ROR*γ*T expression was impaired, Foxp3 levels were increased, and the mice had a defective response to IL-6 [68]. In particular, IRF4 has also been shown necessary for IL-21 to promote further production of itself and expression of IL-23R [69], thus propagating the Th17 positive feedback loop. With regard to T1D, it has been shown that a lack of IL-21/IL-21R signaling confers protection against T1D [73]. BATF−/− mice show a normal cell population distribution but have marked decreases in IL-17 and IL-21 production even while under Th17-inducing conditions. BATF−/− mice were also shown to be resistant to EAE, and although these mice exhibited normal IL-6 and TGF*β* signaling, ROR*γ*T and the *IL17* promoter were shown to have BATF dependence [72]. Notably, reduction in NOTCH signaling also reduces EAE severity [71].

AHR, also referred to as the dioxin receptor, is a ligand-dependent transcription factor whose expression is restricted to the Th17 subset of CD4+ T cells and is found in both mice and humans [70]. AHR deficient naïve T cells possess an impaired capability to differentiate into Th17 cells, even under Th17-inducing conditions [74]. AHR ligation and activation result in dramatic increases in the number of Th17 cells and promote the expression of IL-22, IL-17A, and IL-17F [70]. Interestingly it has been shown that 2,3,7,8-tetrachlorodibenzo-*p-*dioxin (TCDD) and 3-methylcholanthrene (3-MC), two components derived from cigarette smoke and known environmental risk factors for rheumatoid arthritis, act as exogenous ligands for AHR [75]. Thus, AHR may represent an interesting link between environmental factors and autoimmune development in regard to rheumatoid arthritis. Although it is clear that a number of transcription factors contribute to the Th17 phenotype, as the specific effector functions of IL-17-producing cells can vary [76], further research is necessary to determine the specific contribution of each to variations in Th17 effector functions.

Researchers have further explored the link between autoimmunity and environmental factors by looking at the effect of a high salt diet, such as is seen in a typical “Western diet”, in the pathology of autoimmunity, specifically Th17 cells. Kleinewietfeld and colleagues showed that high levels of sodium chloride induce an increase in Th17 cells that were shown to be highly stable and pathogenic [77]. It was determined that increased levels of sodium chloride induce the salt-sensing kinase SGK1, which then upregulates IL-23R expression and Th17 differentiation [78]. Although more research is needed to explore this mechanism, this is an important development as the influence of environmental factors on autoimmune disorder development has become a prominent area of interest.

## 5. Negative Regulation of the Th17 Subset

As with all immunological responses, there are negative regulators set in place in order to better ensure immune homeostasis and prevent aberrant Th17 responses. For example, both Th1 and Th2 cells act as negative reciprocals of each other, and their effector cytokines IFN*γ* and IL-4 act to downregulate the expansion of Th17 cells as well [19, 21, 79]. Interleukin-25 (IL-25), also referred to as IL-17E, is another member of the IL-17 family [80]. IL-25 has been shown to skew immune responses to the Th2 phenotype and was seen to be decreased in the sera and inflamed mucus of patients diagnosed with inflammatory bowel disease (IBD) [81–83]. It has been shown using IL-25−/− mice that the lack of IL-25 increases IL-23 and IL-17 production, therefore resulting in increased EAE disease severity [84]. IL-25 was also shown to inhibit production of TNF*α*, IFN*γ*, and IL-17A but upregulated the production of IL-10 [83].

In T1D studies, mice which received either a neutralizing anti-IL-17 antibody or recombinant IL-25 at 10 weeks of age were able to prevent development of T1D [31]. In the same study, IL-25 was also able to restore normal glucose levels in newly diagnosed NOD mice, delay the return of autoimmunity following syngeneic islet transplant, and reduce autoreactive Th2 and Th17 cell populations to allow for the development of a protective Treg population. IL-25 therefore is a possible target for T1D mediation through regulation of Th17 cells. However, IL-25 is known to bias the immune response towards a Th2 phenotype [80], suggesting that inhibiting Th1 differentiation may play an important role in disease prevention. Moreover, IL-25 is not a Th17-specific cytokine [85] and regardless of the severity of insulitis, the researchers were able to identify only very small numbers of Th17 cells in the pancreas [31]. The upregulation of IL-10 by IL-25 is also likely significant as Th17 cells possess elevated levels of the IL-10 receptor *α* chain. Moreover, numerous studies have shown an important role of IL-10 in the regulation of T1D [86–88]. As Th17 cells are likely involved in the progression of disease severity, but not in the initial onset of T1D, targeting the inflammatory properties of Th17 cells may have efficacy in early onset T1D patients; as this may delay the total ablation of insulin-producing pancreatic *β*-cells and therefore the dependence on exogenously-derived insulin for survival.

IL-27, composed of the Epstein-Barr virus-induced gene 3 (EBI3) and the subunit p28, has also been shown to regulate IL-17 signaling [89]. IL-27 signals through the common gp130 subunit and the IL-27-specific WSX-1 subunit, which is upregulated upon T cell activation [90, 91]. IL-27 suppresses the development of Th17 cells in a STAT1-dependent manner [92, 93] and suppresses IL-6-mediated T cell proliferation, not surprisingly as IL-6 and IL-27 both signal through the shared receptor component gp130 [89, 92]. IL-27 has been shown capable of suppressing the ability of diseased lymph node and spleen cells to confer EAE on healthy mice and was able to suppress autoreactive Th17 cells *in vitro* and *in vivo* [94]. High numbers of Th17 cells and high levels of IL-17 were found in patients with uveitis and scleritis and in murine models of EAU; moreover IL-27 was found to reduce these levels *in vitro* through upregulation of STAT1 [95]. The immune-regulatory properties of IL-27 are also of interest in T1D as GWAS studies have shown deficiencies in both IL-27 and WSX-1 within T1D patient cohorts [96, 97].

IL-2, a cytokine that has been well established as a necessary growth factor for activated T lymphocytes and Tregs, also acts to inhibit Th17 expansion, as blockage of IL-2 or deletion of STAT5 leads to enhanced production of Th17 cells [98]. As IL-2 is critical for the thymic production and peripheral stability of Tregs [99], it is possible that IL-2 deficiency may be acting directly upon the ability of Tregs to regulate the Th17 population. Alternatively, limited amounts of IL-2 may favor the differentiation of naïve T lymphocytes into the Th17 phenotype as opposed to the iTreg. It has also been reported that SOCS3 and SOCS1, members of the protein family of suppressor of cytokine signaling, regulate Th17 differentiation and expansion possibly through modulating IL-23-mediated STAT3 phosphorylation [100, 101]. Notably as cytokines which oppose Th17 differentiation often oppose each other (e.g., IL-2 inhibition by IL-27 or IFN*γ* inhibition by IL-4), [102, 103] more *in vivo* studies must be conducted to further elucidate the mechanisms by which cytokines and the SOCS regulatory pathways moderate IL-17 signaling.

Vitamin D can act as another source of Th17 regulation, as the vitamin D receptor is induced in Th17 cells, and treatment of murine models of EAE with 1,25 Dihydroxyvitamin D3, an active ligand of the vitamin D receptor, ameliorates the disease and reduces the levels of IL-17A and IL-17F [104]. 1,25D3 appears to inhibit Th17 polarization through posttranscriptional regulation, as the suppression of cytokines occurs at the protein level, while mRNA levels remain unchanged [105]. Oral doses of calcitriol, a synthetic vitamin D analog, were also found to prevent and partially reverse uveitis and suppress Th17 induction without affecting transcription factor expression [106].

The importance of vitamin A on immunological and overall health has been long recognized. A metabolite of vitamin A, retinoic acid, has also come into the spotlight as a potent attenuator of immune function and has been shown to have effects on T cell differentiation and function [107]. Early studies indicated that the administration of retinoic acid abrogated EAE development by reducing cellular infiltration and neurological symptoms [108]. Administration of retinoic acid also attenuated an experimental autoimmune model of nephritis, which was associated with decreased antiglomerular basement membrane antibodies, proteinuria, and levels of TNF*α* and IL-1*β* [109]. Retinoic acid production by CD103+ dendritic cells (DCs) was shown to reduce the severity of DSS-induced colitis. In the experimental allergic asthma model, retinoic acid was able to mediate airway inflammation by decreasing Th17 differentiation, IL-17A, production, and ROR*γ*T transcription factor levels [110, 111]. Retinoic acid regulates the inappropriate immune responses found in autoimmune models by inhibiting the differentiation and expansion of the Th17 cell population and increasing Foxp3+ Tregs [110–113]. This effect was seen to be independent of IL-2 or STAT3/STAT5 mechanisms, but it has been suggested that retinoic acid inhibits Th17s and upregulates Tregs by promoting TFG*β*-driven SMAD signaling and blocking the expression of IL-6 and IL-23R [112, 113]. Interestingly 1,25D3 and retinoic acid were found to act synergistically in the inhibition of both human and murine Th17 differentiation and development, along with a decrease in Th17-related cytokines and transcription factors [114].

## 6. Differentiation of Human Th17 Cells

Given the implication of Th17 cells in several murine autoimmune models, it is an important priority to determine whether Th17 cells share a similar pathogenicity in human autoimmune disorders. Although there are distinctions between murine and human Th17 pathways, many similarities still exist. In humans, it has been shown that TGF*β*, IL-1*β*, and IL-6, combined with IL-21 or IL-23 can induce Th17 differentiation [115–118]. Although the role of TGF*β* was first dismissed as dispensable for human Th17 differentiation [115], it has been confirmed to be required for naïve CD4+ T cell differentiation into Th17 cells [116, 117]. Human Th17 cells have also been reported to be promoted by exogenous nitric oxide (NO) and the induction of NOS2 signaling [119]. The transcription factors involved in human Th17 differentiation include RORc (homolog to ROR*γ*T) and STAT3 [116, 120, 121]. Other transcription factors include basic leucine zipper transcription factor, ATF-like (BATF), VDR, NOTCH1, and runt-related transcription factor 1 (RUNX) [71, 122, 123]. NOTCH1 signaling is important in Th17 differentiation, as it regulates ROR*γ*T in both mice and humans and directly interacts with the *IL17* promoter [71]. Runx1 plays a role in Th17 regulation as well through induction of ROR*γ*T expression and suppression of Foxp3 [123].

Similar to mice, human-derived Th17 cells produce a group of cytokines including IL-17A, IL-17F, IL-21, IL-22, TNF*α*, and IFN*γ*, in addition to IL-26 and CCL20 (whose ligand is CCR6) [118, 124, 125]. Human Th17 cells are readily identified by surface cell markers including CCR6 and CCR4 [120]. Notably, it has been shown that human Th17 cells originate from CD4+CD161+ T cell precursors and that all IL-17-producing cells were contained in the CD161+CCR6+ T cell subset and are also able to express IL-23R [124].

The IL-17 receptor is found on a wide variety of cells including hematopoietic cells, osteoblasts, fibroblasts, endothelial cells and epithelial cells and when bound to IL-17 can induce the release of inflammatory proteins including IL-1*β*, IL-6, IL-8, IL-23, TNF, ICAM-1, PGE_2_, and GM-CSF [126–130]. GM-CSF governs the growth and differentiation of granulocytes and macrophages, thereby playing a crucial role in the innate inflammatory response [131]. Moreover, the prostaglandins produced serve to perpetuate the Th17 inflammatory cycle. Prostaglandins, which are hormone-like lipid compounds derived from fatty acids, are active in promoting inflammation in the tissues of both humans and mice [132] and have been shown to induce IL-23 production in bone marrow-derived dendritic cells and increase p19 and p40 (but not p35) expression through EP2/EP4 receptors [133, 134]. PGE_2_ is also capable of inducing IL-1*β* and IL-6 in an IL-23-dependent manner and also CCL20, MIP-3*α*, CXCL8/IL-8, and CCR6 [133–135].

## 7. Functions of the Th17 Lineage

Th17 cells have earned a well-deserved reputation as key modulators in autoimmunity; however, the Th17 lineage has pleiotropic effects on a variety of cell types including epithelial, endothelial, and fibroblastic cells [130]. The Th17 lineage provides a unique mechanism for protection against bacterial and fungal pathogens through production and induction of inflammatory cytokines and other proteins. The Th17 lineage is also largely responsible for the induction of granulopoiesis, or the recruitment of neutrophils, a vital and key component of the innate immune response [136]. For example, mice deficient in IL-17R, therefore lacking the ability to recruit the necessary numbers of neutrophils, experience 100% mortality when challenged with *Klebsiella pneumoniae* [137]. Neutrophil recruitment induced by Th17 cells is also necessary for pathogen protection and clearance in several other models including *Mycoplasma pneumoniae*, *Bordetella pertussis*, *Candida albicans*, *Pneumocystis carinii*, *Francisella tularensis*, *Staphylococcus aureus,* and *Citrobacter rodentium* [138–146]. However clearance of *Mycobacterium tuberculosis* and intracellular bacteria such as *Salmonella enterica* are more reliant on a Th1 response, as Th17 immune responses are only slightly effective in the absence of the Th1 pathway [147–149]. Th17 cells have also been found necessary for complete vaccination protection against the 3 systemic mycoses that are endemic to North America which include *Coccidioides posadasii, Histoplasma capsulatum, and Blastomyces dermatitidis* [150, 151].

## 8. A Proinflammatory Role for Th17 Cells in Autoimmune Disorders

Since the identification of the Th17 lineage, researchers have continued to garner information on the role of Th17 cells in not only murine models of disease, but also from human samples as well. In the following section we will discuss the role that Th17 cells play in the mentioned murine models and the human disease counterparts.

As mentioned previously, Th17 cells were found to be the central effector lineage involved in the pathogenicity of murine EAE, an important model of multiple sclerosis (MS) [11]. MS is a neurodegenerative autoimmune disorder in which axon demyelination lesions develop in the central nervous system (CNS) [90]. Prior to the discovery of Th17 cells, researchers had already begun to publish on the positive correlations found between levels of IL-17, IL-6, and G-CSF and the progression of active multiple sclerosis [152]. With the relatively recent discovery of T helper 17 cells, research disseminating its newly described role in EAE and MS progressed quickly. Human blood-brain barrier endothelial cells were found to express receptors for IL-17 and IL-22, thus making it possible for IL-17 and IL-22 to disrupt blood-brain barrier junctions [153]. It was also found that human Th17 lymphocytes were able to migrate past blood-brain barrier-associated cells, where they continued to promote inflammation through CD4+ T cell recruitment and inflammatory cytokine production [153]. Further studies continued to correlate the number of Th17 cells and amount of IL-17 expression found in MS lesions with active disease [154, 155]. Researchers observed that IFN-*β* and IL-27 were able to suppress active disease in MS patient-derived cells and in murine EAE, which is consistent with previous knowledge of their Th17-suppressive capabilities [156].

With regard to the role of microbiota in the prevention or progression of EAE and MS, reports have thus far been varied. Researchers have shown that the use of specific probiotic mixtures is able to suppress EAE development though Th1/Th17 polarization inhibition, and increases Foxp3+ Treg numbers and IL10 production [157]. Still, others have also described a pathogenic role for gut microbiota including several studies which showed that germ-free mice or antibiotic-treated mice had reduced disease severity, reduced proinflammatory cytokine productions, and an increase in Treg accumulations [158]. Yokote and colleagues also demonstrated that the administration of antibiotics altered gut flora composition and ameliorated EAE development through a possible invariant natural killer cell-Th17 interaction-dependent mechanism [159].

The etiology of psoriasis, a skin-associated inflammatory disease, has also shifted from a Th1 to a Th17 focus [160]. The idea that psoriasis was a Th1-regulated autoimmune disorder was supported by studies such as the one done by Kauffman et al. in 2004, which found success during a phase 1 clinical trial using a humanized monoclonal antibody to IL-12p40. Patients improved significantly; however, without knowing that IL-23 and IL-12 share the p40 subunit, researchers attributed its success to Th1 inhibition [161]. Data began to show, however, that, while infiltrative and activated T cells are the primary modulators of disease progression, Th1 cells may not be as centrally involved as originally thought [162]. When dendritic cells are activated by inflammatory stress signals from keratinocytes (the predominant cell type in the epidermis), DCs can actively skew naïve CD4+ T cells into subsets of specific T helper cells, especially Th17 cells [163]. Once Th17 cells have differentiated and localized to the tissue site of inflammation, their CCR6 ligand is able to bind to CCL20 found on local keratinocytes [164]. The production of inflammatory cytokines from these localized Th17 cells can upregulate CCL20 expression from antigen presenting cells and CCR6 expression on the Th17 cells, thus creating a system which propagates the Th17 population [164]. To further confirm the vital role of the Th17 subset in psoriasis, patients have been shown to have increased levels of CCL20, CCR6, and Th17 cells in the psoriatic lesions compared to healthy controls [165].

Rheumatoid arthritis (RA) is an autoimmune disorder whose symptoms include synovial inflammation, autoantibody production, and cartilage and bone destruction among a list of others [166]. A common mouse model for RA is the collagen-induced arthritis (CIA) model, whose pathogenicity was originally attributed to a deregulated T helper 1 inflammatory process [76]. In 2003 Murphy et al. saw that IL-23p19−/− mice were protected from CIA, whereas IL-12p35−/− mice exhibited an exacerbated disease [18]. Blockage of IL-23 expression protected mice from joint and bone destruction, and an anti-IL-17 antibody has been shown to inhibit osteoclast formation in human rheumatoid arthritis samples [18, 167]. IFN*γ* was in actuality shown to be capable of negatively regulating Th17 differentiation and suppressing CIA through the induction of indoleamine-2,3-dioxygenase, a compound which has previously been shown capable of regulating autoreactive Th17 cells [168]. PGE_2_, a known positive regulator of Th17 differentiation, was shown capable of enhancing CIA severity through enhanced DC-derived IL-6 production, thereby shifting the IL-23/IL-12 more towards IL23 and IL17 [169]. Patients with RA exhibit high levels of IL-17, IL-17R, IL-1*β*, and IL-6 among others in synovial fluid samples [167, 170, 171]. IL-17 has also been found in large quantities in the synovial biopsies of RA patients [171]. IL-17 was shown to augment production of nitrous oxide, which has previously been described to promote RA-associated autoinflammation [172].

Much like in MS, the potential role that an individual's microbiota might have in RA disease development or prevention is of high interest. In a study of streptococcal cell wall-induced arthritis in F344 rats (which are resistant to chronic joint inflammation), germ-free F344 rats developed SCW-induced arthritis, while conventionalization dramatically moderated the arthritic phenotype [173]. However in another study using HLA-B27 transgenic rats, it was shown that rats in the germ-free state were protected against chronic inflammation including colitis and arthritis, while transgenic conventionalized rats developed high levels of proinflammatory cytokine production. Thus the normal luminal bacteria induced systemic inflammation [174, 175]. Notably, a study by Wu and colleagues revealed that germ-free conditions protected mice from arthritis development as seen with reductions in Th17 populations, while neutralization of IL-17 in SPF mice also had similar effects. It was also shown in the same study that a single, gut-residing species segmented filamentous bacteria restored Th17 induction and subsequent arthritis development [176].

Inflammatory bowel disease (IBD) is comprised of Crohn's disease (CD) and ulcerative colitis (UC), and results when the mucosal immune system mounts an inappropriate and sustained inflammatory response against normal, resident gut flora [177]. The relative success of an anti-IL-12 antibody in ameliorating CD again encouraged researchers to classify colitis as a product of a Th1 immune response [178]. While there have been conflicting results published as to whether Th17 cells play a pathogenic or protective role in IBD, there is very strong evidence to support the former. IL-23p19, IL-17, and IRF4 deficient mice were both protected from colitis-related symptoms, including weight loss and production of high levels of proinflammatory cytokines including IL-6, IL-17, and IL-22 [179–181]. The role of Th17 cells in colitis is also supported in reports which indicate that PGE_2_ augments expansion of the Th17 population by skewing DC production of IL-12 to IL-23 [182]. Development of colitis has been shown to be inhibited by blocking production of IL-6, which encourages apoptosis of lamina propia autoreactive Th17 cells [183]. A humanized anti-IL-6 monoclonal antibody has also showed promising results in a pilot trial involving patients with active disease [184]. A genome-wide association study with CD patients showed a high association of IL-23R and CCR6 to states of active disease, while another study indicated that patients with active disease had higher numbers of IL-17 and IL-23p19+ cells in the lamina propria and had increased mRNA expression of IL-17, IL-6, IL-23, IL-1*β*, and CCR6 [185–188]. In addition, patients have also been found to have higher levels of IL-17 in sera and inflamed mucosa samples, as well as increases in pSTAT3 and NOS signaling [189].

With regard to IBD, a molecular-based analysis of microbiome populations in the small intestines of CD and UC patients revealed significant differences in the makeup of sick patients compared to those of healthy controls [190]. Decreases in members of *Firmicutes* and *Bacteroidetes* represented significant abnormalities in patients with IBD [190]. Studies involving monozygotic twins revealed that healthy individuals had a higher bacterial diversity than twins with CD and that the intestinal microbial compositions differed between healthy and sick individuals [191, 192]. A study done by Frank et al. in 2011 showed that markers of IBD phenotype and genotype correlated with shifts in intestinal-dwelling microbiota [193].

Another disease exhibiting strong links to the Th17 lineage is systemic lupus erythematosus (SLE). SLE is a complex autoimmune disorder characterized by autoantibody and immune complex formation and has has been strongly correlated to a dysregulated Th17/regulatory T cell balance [194, 195]. IL-17 and Th17 cells have been detected at higher levels in several murine models of lupus compared to those found in healthy, wild type mice [196]. A role for Th17 cells in murine lupus was demonstrated with the success of an anti-CD3 antibody in symptom improvement whose effects were dependent on decreased IL-17 production and a decrease in Th17 kidney-infiltrating cells; tolerance was correlated with decreased levels of IL-6 production but an increase in TGF*β* and regulatory T cells [197]. Overexpression of the Epstein-Barr virus-induced gene 3 (EBI3) in an MRL/lpr murine lupus model resulted in relatively normal renal function, decreases in anti-dsDNA, and an increase in Foxp3+ Tregs, supporting the need for a correct regulatory T cell/Th17 ratio [198].

It has been proposed in SLE that DCs commonly come into contact with apoptotic debris and therefore develop into a mature phenotype and produce higher amounts of IL-6; in turn the higher level of IL-6 production results in the differentiation of naïve CD4+ T cells into Th17 cells, when in fact they may have been converted into regulatory T cells under normal conditions [199]. SLE patients have elevated levels of inflammatory cytokines including IL-23, IL-17, and IL-6 and higher frequencies of lymph nodal Th17 cells [200, 201]. SLE patients have also been found to have decreased absolute numbers of regulatory T cells, and it has been shown that higher numbers of regulatory T cells correlate to decreased disease severity [202].

Another piece of evidence that supports the role of Th17 cells in lupus pathology is the use of hydroxychloroquine in the treatment of SLE patients [203]. Hydroxychloroquine in an antimalarial agent, which has recently been shown to ameliorate SLE symptoms by inhibiting the production of IL-6, IL-17, and IL-22 possibly through reduction of the Th17 population [203].

Asthma is a chronic lung disease associated with airway inflammation, airway hyperresponsiveness, increased mucus production, and infiltration by eosinophils and T lymphocytes [204]. It has generally been accepted that Th2 cells as well as the Th2-related cytokines IL-4, IL-5, and IL-13 play a central role in eosinophil infiltration into the airways and the resulting pathogenicity of the disease [205]. Recent research indicates that in addition to Th2 cells, Th17 cells and IL-17 may also be playing a critical role in asthma development and progression [204]. In mice sensitized with ovalbumin (a classical method by which to study asthma pathology), IL-17 mRNA expression was elevated in inflamed lung tissue and was correlated to enhanced neutrophil recruitment to the airways. In this same study, an anti-IL-17 monoclonal antibody significantly reduced the level of neutrophil influx into these tissues [206]. Similar to IL-17A, IL-17F has also been shown to increase neutrophil recruitment and levels of CXC chemokine and inflammatory cytokine gene expression [207]. A common treatment for asthmatic symptoms is the administration of corticosteroids to reduce the airway inflammation. Th17 cells however are involved in mediating what has been termed “steroid-resistant” airway inflammation [205, 208]. Several studies have shown that, while Th2-mediated inflammation and eosinophil infiltration respond to the administration of steroids, neutrophil influx and inflammatory Th17 cytokines are nonresponsive [208]. Kobayashi et al. have also recently reported that IL-1 family member, IL-1*β*, may potently induce Th17 differentiation in response to airborne antigens [209].

The number and percentages of Th17 cells in the sputum, lung tissue sections, and submucosa of asthma patients are also elevated compared to those of healthy controls [210, 211]. Fibroblasts and macrophages from asthmatic patients generated more GM-CSF, TNF*α*, IL-1*β*, IL-8, and IL-6 *in vitro* when stimulated with IL-17 [212]. Interestingly exposure to diesel exhaust particles (which are a major component of air pollution caused by traffic) not only led to Th17 cell accumulation in the lungs of mice, but also increased asthmatic symptoms and IL-17A serum levels in children [213].

The role of microbiota in asthma has also been explored, and Russell et al. have reported that treatment of neonatal mice with antibiotics results in susceptibility to allergic asthma following a shift in the resident gut flora [214]. In a following study by Russell et al. it was shown that this antibiotic driven shift in microbiota resulted in increased IgE production and decreased Tregs. The role of Th17 cells in the development of asthma has only recently been described, and requires more in-depth analysis.

## 9. A Protective Role for Th17 and Gut Flora in T1D

Type 1 diabetes is one of the most prevalent autoimmune diseases in modern day society, with over 1 million Americans having been diagnosed and 15,000 more children being diagnosed each year [215]. T1D is a CD4+ and CD8+ T-cell-mediated autoimmune disorder which targets and destroys insulin producing *β*-cells in the pancreas. The absence of insulin results in uncontrolled blood glucose levels, leading to clinical symptoms which can include excessive thirst, frequent urination, tissue dehydration, and weight loss [216, 217]. As pancreatic *β*-cells do not normally possess surface molecules sufficient to activate naïve T lymphocytes [218], it has been postulated that conventional T cell receptor (TCR)/MHC/antigen-mediated activation is occurring through interactions with APCs sampling antigens from the pancreas. The APCs, such as dendritic cells, present pancreatic antigens to T cells in the pancreatic lymph node (PLN), which leads to increased development of autoreactive T cells [219–221]. This is supported by the fact that removal of PLN from young nonobese diabetic (NOD) mice, a classical model of T1D, prevents onset of T1D [222].

Although genetic predisposition is critical in T1D onset [223], a concordance rate between monozygotic twins of 30–50% [224–226] implies that environmental factors also play a significant role. When discussing the effect of environmental cues on health and disease, the gut microflora of each individual, in addition to diet and chemical exposure, is undoubtedly an important factor to be considered. The human body plays host to as many as 100 trillion bacteria in the digestive tract alone, thus ensuring a continued interaction between these resident microbes and the mucosal immune system [227]. Gut microbiota provide several important functions including the absorption of nutrients and protection against pathogenic bacterial species [228]. Studies have shown that alterations of the gut composition through Caesarian methods, germ-free environments, or antibiotic usage resulted in changes in T1D onset and progression, clearly indicating the important effect gut composition has on disease modulation and health [229–231]. A gut microbiome metagenomics analysis indicated that autoimmune subjects have a functioning aberrant microbiome [232], and children with *β*-cell autoimmunity, indicative of T1D, have a markedly different composition of gut flora species than do healthy controls [233]. The capability of lamina propia DCs to differentiate CD4+ T cells into Tregs in T1D patients is impaired, which suggests a possible lack of intestinal immunoregulation by gut flora [234]. Moreover, the association of Th17 lymphocytes with T1D progression has been heightened by the importance of Th17 cells in the regulation of commensal bacteria and extracellular bacterial pathogens [137, 139, 235].

**Figure 2:**
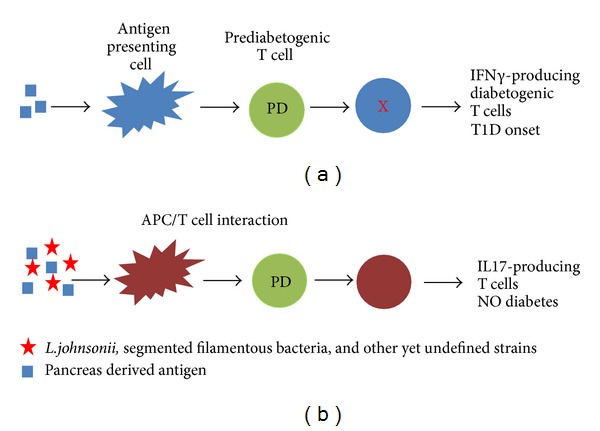
A Proposed model of bacterial regulation of T1D. Type 1 diabetes is due to the acquisition of diabetogenic effector functions by prediabetogenic T lymphocytes (T lymphocytes which bear TCR specific for pancreas related antigens have not yet been activated). Gut flora has been shown to play a critical role in immune homeostasis (see text). In this model we propose that specific microbial strains such as *L. johnsonii* and SFB, which naturally reside within the mucosa, can inhibit the onset of T1D by inhibiting the development of diabetogenic effector functions by T lymphocytes. While IFN*γ* has been associated with effector functions which lead to T1D, the work by us and others has implicated that IL17 production by T lymphocytes is associated with effector functions that are protective in T1D.

Several studies, including our own, have highlighted the importance of how changes in gut flora modulate the mucosal immune response, specifically in T1D onset and progression [31]. In a recent study it was shown that the transfer of gut microbiota from healthy adult male NOD mice to immature NOD females altered the females' gut microbiome resulting in reduced islet inflammation, reduced autoantibody production, and vigorous protection against T1D [236]. Biobreeding diabetes prone (BB-DP) and biobreeding diabetes resistant (BB-DR) rats are models that can provide insight into gut microflora in the context of T1D [237]. When BB-DP rats were fed *Lactobacillus johnsonii* N6.2 (LjN6.2) and *Lactobacillus reuteri* isolated from BB-DR rats, diabetes onset was inhibited with LjN6.2, while the addition of *L. reuteri* increased incidence of disease [238]. Interestingly, it was shown that BB-DP rats exhibiting T1D resistance possessed a larger population of Th17 cells in the mesenteric lymph nodes and spleen compared to their sick counterparts [239]. LjN6.2 was further shown to be capable of upregulating Th17 populations *in vitro* as well [239]. Moreover, we showed that dendritic cells, incubated with LjN6.2, were sufficient to mediate a Th17 bias both *in vitro* and *in vivo*. Although NOD mice are genetically prone to develop T1D, it was shown that NOD mice that were resistant to T1D had a natural segregation of segmented filamentous bacteria that also mediated a Th17 bias [240]. The studies outlined in this review present a model whereby gut flora-mediated Th17 differentiation rescues T lymphocytes from a diabetogenic phenotype. Moreover IL-17 production could potentially serve as a biomarker indicating T lymphocytes that were inhibited from acquiring diabetogenic properties. This finding is potentially significant as comparing gut flora-modulated Th17 cells to “diabetogenic” T lymphocytes could assist in identifying true effector functions leading to T1D onset (Figure 2).

## 10. Discussion

Considerable progress has been made in our understanding of IL-17-producing, CD4+ T lymphocytes since their initial discovery. Scientists have progressed rapidly in our knowledge of Th17 differentiation, regulation, and its role in autoimmunity. It has become increasingly clear that the interface between Th17 cells and the native gut flora play a crucial role in the immune response [228, 241]. With that in mind, there is ample data to suggest the involvement of gut flora composition in modulation of EAE [157, 159, 242], IBD [190–193], arthritis [173–176], and asthma [214, 242]. This would suggest that a dysbiosis of the microflora may drive aberrant Th17 activity and thereby play an important role in the progression of autoimmune disorders.
